# 17q23.2q23.3 de novo duplication in association with speech and language disorder, learning difficulties, incoordination, motor skill impairment, and behavioral disturbances: a case report

**DOI:** 10.1186/s12881-017-0479-3

**Published:** 2017-10-25

**Authors:** Karen Wessel, Jehan Suleiman, Tamam E. Khalaf, Shivendra Kishore, Arndt Rolfs, Ayman W. El-Hattab

**Affiliations:** 1 Centogene AG, Schillingallee, Rostock, Germany; 2 Division of Neurology, Department of Pediatrics, Tawam Hospital, Al Ain, United Arab Emirates; 3 Biochemistry, University of Toronto Mississauga, Mississauga, Canada; 4 Albrecht-Kossel-Institute for Neuroregeneration, Medical University Rostock, Gehlsheimer Straße, Rostock, Germany; 5 Division of Clinical Genetics and Metabolic Disorders, Department of Pediatrics, Tawam Hospital, Al-Ain, United Arab Emirates

**Keywords:** 17q23.2q23.3 duplication, Genomic rearrangements, Chromosomal microarray, Chromosomal disorders

## Abstract

**Background:**

Chromosomal rearrangements involving 17q23 have been described rarely. Deletions at 17q23.1q23.2 have been reported in individuals with developmental delay and growth retardation, whereas duplications at 17q23.1q23.2 appear to segregate with clubfoot. Dosage alterations in the *TBX2* and *TBX4* genes, located in 17q23.2, have been proposed to be responsible for the phenotypes observed in individuals with 17q23.1q23.2 deletions and duplications. In this report, we present the clinical phenotype of a child with a previously unreported de novo duplication at 17q23.2q23.3 located distal to the *TBX2* and *TBX4* region.

**Case presentation:**

We report a 7.5-year-old boy with speech and language disorder, learning difficulties, incoordination, fine motor skill impairment, infrequent seizures with abnormal EEG, and behavior disturbances (mild self-inflicted injuries, hyperactivity-inattention, and stereotyped hand movements). Chromosomal microarray revealed a 2-Mb duplication of chromosome 17q23.2q23.3. Both parents did not have the duplication indicating that this duplication is de novo in the child.

**Conclusions:**

The duplicated region encompasses 16 genes. It is possible that increased dosage of one or more genes in this region is responsible for the observed phenotype. The *TANC2* gene is one of the genes in the duplicated region.It encodes a member of the TANC (tetratricopeptide repeat, ankyrin repeat and coiled-coil containing) family which includes TANC1 and TANC2. These proteins are highly expressed in brain and play major roles in synapsis regulation. Hence, it is suggestive that *TANC2* is the likely candidate gene responsible for the observed phenotype as an increased *TANC2* dosage can potentially alter synapsis, resulting in neuronal dysfunction and the neurobehavioral phenotype observed in this child with 17q23.2q23.3 duplication.

## Background

The expanded use of chromosomal microarrays has significantly improved the yield of diagnosing genomic disorders and led to the identification of several novel microdeletion and microduplication syndromes [1, 2]. Chromosomal microarrays, which have been routinely used as a first-tier diagnostic test for individuals with neurodevelopmental disabilities or congenital anomalies, can detect pathogenic copy number variations with a diagnostic yield of 10–20% compared to the 3% karyotyping diagnostic yield [1, 3, 4].

Chromosomal rearrangements involving 17q23 have been described rarely. Seven individuals were reported with 2.2–2.8 Mb deletions at 17q23.1q23.2 [5]. All these individuals had mild to moderate developmental delay. Additional features were microcephaly, growth retardation, heart defects, hand and foot anomalies, musculoskeletal abnormalities, behavioral abnormalities, hearing loss, and some distinctive facial features. Parental testing in five of the seven cases confirmed a de novo origin of the deletions [5]. A 2.2 Mb duplication at 17q23.1q23.2 identified in 10 individuals from 3 families was found to segregate with autosomal-dominant clubfoot in these families but with reduced penetrance [6]. Mild short stature and other skeletal abnormalities were also commonly observed in these individuals [6]. Dosage alterations in the *TBX2* and *TBX4* genes, located in 17q23.2, have been proposed to be responsible for the phenotypes observed in individuals with 17q23.1q23.2 deletions and duplications as these genes encode transcription factors implicated in a variety of developmental pathways [5, 6].

In this report, we present a child with speech and language disorder, learning difficulties, incoordination, fine motor skill impairment, and behavior disturbances who was found to have a previously unreported de novo duplication at 17q23.2q23.3 located distal to *TBX2* and *TBX4* region. Herein, we describe the details of the clinical phenotype, present the results of the molecular tests, and discuss the genes in the duplicated region.

## Case presentation

A 7.5-year-old boy was born at term via normal vaginal delivery following an uncomplicated pregnancy. His birth growth parameters were normal and he had normal newborn physical examination. He developed urinary tract infection at the age of 40 days and was found to have bilateral hydronephrosis and vesicoureteric reflux. Prophylactic antibiotics were given during the first 2 years of life after which hydronephrosis and reflux resolved. During early childhood, his parents were concerned as he had delayed speech. He started his first words at the age of 3 years. His speech improved but remains impaired and not fully intelligible. He had good communication and social interaction including non-verbal communication. His receptive language was better than the expressive language; however he had difficulty with comprehension of complex commands or conversations. His early motor development was normal. At the time of this report he was able to run; however, he trips and falls frequently. He was unable to skip or jump, and needed to hold onto the rail while going up and down stairs. In addition, he had difficulties with fine motor skills including writing. These symptoms likely caused by incoordination. He had reasonable self-care skills including dressing and feeding and was toilet trained by age 3.5 years. The boy had behavior disturbances in the form of mild self-inflicted injuries (scratching his skin and attempting to peel his nails), and hyperactivity-inattention that had improved with atomoxetine. The child attended school and had difficulties in learning including reading and writing. A formal IQ test was performed at 7 years of age using Stanford-Binet Intelligence Scales Fifth Edition (SB5) and revealed low-average scales (85–93). The child had one brief generalized tonic-clonic seizure at the age of 6.5 years, and no antiepileptic medications were initiated. He remained free of seizures after that. Family history was notable of parental consanguinity. The child had a brother and a sister who had been healthy.

Physical examination revealed normal growth parameters including head circumference and distinctive facial features including thick eyebrows, down-slanted palpebral fissures, low-set posteriorly rotated ears, and thick lower lip. He was able to follow simple commands and to engage in simple conversation. His speech was largely non-intelligible. Stereotyped repetitive hand movements were noted. Cranial nerve examination was normal. His muscle tone and power, deep tendon reflexes, and sensory exam were normal. He had difficulties with fine finger movements and writing. In addition, he had difficulties with balancing on one foot and with tandem walking.

An electroencephalogram (EEG) at age 6.5 years following the single seizure episode showed relatively slow background (theta range) and no epileptiform discharges, a repeat EEG at 7 years showed posterior dominant rhythm of mixed alpha and theta activity with superimposed fast beta in the frontal regions, normal sleep architecture, and infrequent high amplitude epileptic discharges in right frontocentral area during sleep.

Inborn errors of metabolism and chromosomal abnormalities were considered. Metabolic work up (ammonia, lactate, plasma amino acids, urine organic acids, homocysteine, and acylcarnitine profile), thyroid function, karyotype, hearing assessment, cardiac echocardiogram, renal ultrasound (at the age of 2 years), and cranial MRI (at the age of 7 years) were all normal.

Chromosomal microarray revealed a 2-Mb duplication of chromosome 17q23.2q23.3 encompassing Ch17:59,678,856–61,679,670 (hg19). Chromosomal microarray for both parents did not reveal the duplication indicating that this duplication is de novo in the child. The chromosomal microarray (CentoArrayCyto™- HD) was performed at Centogene AG, Schillingallee, Rostock, Germany. DNA was fragmented, amplified and hybridized to the array according to manufacturer’s guidelines. The Cytoscan HD array (Affymetrix) contains 2.7 million markers, including 750,000 SNP markers, across the whole genome covering 96% of the genes. It enables the detection of copy number variations and/or large deletions/duplications. The results were analyzed with the Chromosome Analysis suite (ChAS, Affymetrix). Copy number variations with a minimum of 25 markers and a size of more than 50 kb (deletions) and 200 kb (duplications) were considered. The results were interpreted using the DGV and Decipher databases and additional available databases.

The duplicated region encompasses the *BRIP1* gene at the proximal end and the *TACO1* gene at the distal end. To confirm the duplication with orthogonal methods, multiplex ligation-dependent probe amplification (MLPA) and quantitative PCR assay (qPCR) were used. MLPA analyses were performed using SALSA MLPA P240 BRIP1/CHEK1 provided by MRC-Holland to test for deletions or duplications within or including the *BRIP1* gene. This probemix, which contains probes for each 20 exons of *BRIP1*, demonstrated duplication of all probes against the *BRIP1* gene, suggesting the breakpoint is located proximal to the *BRIP1* gene. For the *TACO1* gene, qPCR assay was performed using VeriQuest Fast SYBR Green (Affymetrix). Three gene specific amplicons targeting exon 1, intron 1 and exon 2 of *TACO1* were used to determine the regions duplicated within the gene. Only probes against exon 1 and intron 1 showed duplication suggesting that exon 2 was not a part of the duplicated region and the duplication breakpoint is located in intron 1. The CARE guidelines were followed in reporting this case.

## Discussion and conclusions

In this report, we describe a 2-Mb duplication at 17q23.2q23.3 in a child with speech and language disorder, learning difficulties, incoordination, fine motor skill impairment, infrequent seizures with abnormal EEG, behavior disorders (mild self-inflicted injuries, hyperactivity-inattention, and stereotyped hand movements), and distinctive facial features (thick eyebrows, down-slanted palpebral fissures, low-set posteriorly rotated ears, and thick lower lip). This duplication has not been previously reported; however, an overlapping duplication at 17q23.1q23.2 has been reported to be associated with clubfoot and other skeletal manifestations, in the absence of cognitive or behavioral impairments [6].

**Fig. 1:**
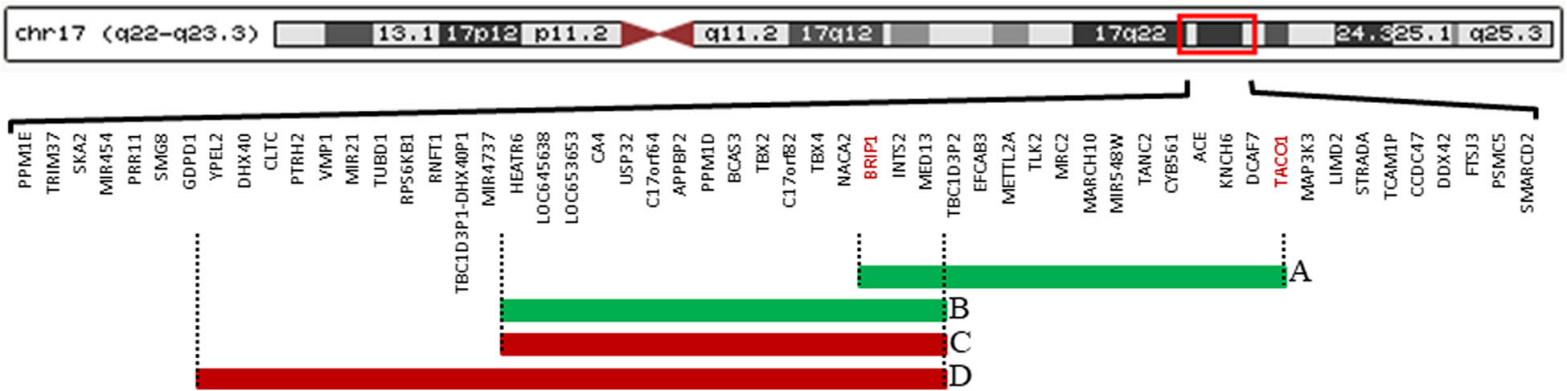
Diagram depicting the organization of genes in the 17q23 regions and the genomic arrangements involved within region. The structure of this region has been simplified for illustrative purposes. **A:** The 2 Mb duplication at 17q23.2q23.3 in the child described in this report. **B:** The 2.2 Mb 17q23.1q23.2 duplication in families described by Alvarado and colleagues [6]. This duplication was associated with clubfoot, mild short stature, and other skeletal abnormalities [6]. **C:** The 2.2 Mb 17q23.1q23.2 deletion in patients 1, 3, 4, 5, 6, and 7 described by Ballif and colleagues [5]. **D:** The 2.8 Mb 17q23.1q23.2 deletion in patient 2 described by Ballif and colleagues [5]. The 2.2 and 2.8 Mb 17q23.1q23.2 deletions were associated with developmental delay, microcephaly, growth retardation, heart defects, hand and foot anomalies, musculoskeletal abnormalities, behavioral abnormalities, hearing loss, and some distinctive facial features [5]

The 17q23.2q23.3 duplication in this child was found to be de novo and was confirmed by MLPA and qPCR. It is possible that increased dosage of one or more genes in the duplicated region is responsible for the observed phenotype. The duplicated region encompasses 16 genes, of which 2 are well-established to be associated with human diseases, the *BRIP1* linked Fanconi anemia and breast cancer susceptibility, and the *TACO1* genes linked to complex IV deficiency [7–9] (Fig. 1). *TACO1* encodes a mitochondrial translational activator required for efficient translation of subunit I of cytochrome c oxidase (complex IV). Biallelic *TACO1* mutations cause mitochondrial complex IV deficiency [9]. It is unlikely that *TACO1* plays any role in the phenotype of this child because it is associated with an autosomal recessive disease caused by biallelic loss-of-function mutations. Furthermore, as the breakpoint is localized within *TACO1* the duplication will result in an intact copy of the gene and a disrupted one similar to the effect of int22h1/int22h2-mediated Xq28 duplication on *F8* gene [10]. Therefore, this 17q23.2q23.3 duplication in not expected to alter the *TACO1* gene dosage.

*TANC2* is another gene in the duplicated region that can potentially be associated with human disease. This gene was reported as a candidate intellectual disability gene in one study which performed whole exome sequencing for a large cohort of individuals with intellectual disability and identified a de novo missense mutation in *TANC2* in one boy with developmental delay [11]. *TANC2* encodes a member of the TANC (tetratricopeptide repeat, ankyrin repeat and coiled-coil containing) family which includes TANC1 and TANC2. These proteins have several domains for protein-protein interaction, highly expressed in brain, and play major roles in synapsis regulation. Overexpression of TANC1 and TANC2 in cultured neurons appears to increase the density of dendritic spines and excitatory synapses. On the other hand, TANC1-deficient mice exhibit reduced dendritic spine density and TANC2 deficiency causes embryonic lethality suggesting important implication of TANC2 in embryonic development [12, 13]. These studies do not only support the role of TANC in nervous system development and function, but also provide strong evidence that these genes are dosage-sensitive. Increased dosage of other genes in the duplicated region has not been implicated in human diseases. Therefore, it is tempting to speculate that *TANC2* is the likely candidate gene responsible for the observed phenotype associated with this duplication as increased dosage of the *TANC2* gene can potentially alter the synapsis, resulting in neuronal dysfunction and the neurobehavioral phenotype observed in this child with 17q23.2q23.3 duplication. This suggestion is further supported by the notion that the previously reported overlapping duplication at 17q23.1q23.2 that segregated with clubfoot did not include the *TANC2* gene and was not associated with cognitive or behavioral problems [6].

This is the first child to be reported with 17q23.2q23.3 duplication. Being de novo and harboring potential dosage sensitive genes are suggestive that this duplication is responsible for the observed phenotype. However, additional cases are needed to confirm the pathogenicity of this duplication and to further delineate the clinical phenotype associated with it.

In summary, we report a de novo 17q23.2q23.3 duplication in a child with speech and language disorder, learning difficulties, incoordination, fine motor skill impairment, infrequent seizures, and behavior disorders. It is possible that increased dosage of one or more genes in this region is responsible for the observed phenotype. The *TANC2* gene within the duplicated region encodes a member of the TANC family which is highly expressed in brain and plays major roles in synapsis regulation. Consequently, it is likely that *TANC2* plays a role in the observed neurobehavioral phenotype in this child with 17q23.2q23.3 duplication.
